# Sodium Alginate Based Mucoadhesive System for Gatifloxacin and Its *In Vitro* Antibacterial Activity

**DOI:** 10.3797/scipharm.1004-24

**Published:** 2010-09-26

**Authors:** Karthikeyan Kesavan, Gopal Nath, Jayanta K. Pandit

**Affiliations:** 1 Department of Pharmaceutics, Institute of Technology, Banaras Hindu University, Varanasi, Uttar Pradesh – 221 005, India; 2 Department of Microbiology, Institute of Medical Science, Banaras Hindu University, Varanasi, Uttar Pradesh – 221 005, India

**Keywords:** Gatifloxacin, Mucoadhesive, Mucin, *In vitro* release, *In vitro* antibacterial study, Rheology

## Abstract

The objective of this study was to formulate sodium alginate based ophthalmic mucoadhesive system of gatifloxacin and its *in vitro* antibacterial potential on pathogenic microorganisms, *Staphylococcus aureus* and *Escherichia coli*. Sodium carboxymethylcellulose (NaCMC) was added to the formulations to enhance the gel bioadhesion properties. The prepared formulations were evaluated for their in vitro drug release, gelation behaviour, rheological behavior, and mucoadhesion force. All formulations in non-physiological and physiological condition showed pseudo plastic behavior. Increase in the concentration of sodium alginate and sodium CMC enhanced the mucoadhesive force significantly. *In vitro* release of gatifloxacin from the system in simulated tear fluid (STF, pH – 7.4), was influenced significantly by the properties and concentration of sodium alginate, NaCMC. Significant reduction in total bacterial count was observed between control and treatment groups with both the test organisms.

## Introduction

The design of an ocular dosage form which prolongs the precorneal residence time of the drug and enhances the corneal permeation is an exciting challenge. Ocular therapeutic preparations like ophthalmic solutions, suspensions and semisolids are characterised by several important disadvantages. Protective mechanisms including reflex blinking, solution drainage, lachrymation and a highly selective corneal barrier allow only 1–10% of the topically applied dose to be absorbed [1].

Different kinds of approaches can be employed to increase the ocular bioavailability of the drug instilled, to extend the drug residence time in the cul-de-sac and to reduce the probability of adverse systemic effects. Incorporation of soluble polymers into an aqueous solution can be applied to extend the drug residence time, thereby prolonging drug absorption. Solutions of equal viscosity induce the same magnitude of increase in corneal drug absorption regardless of the chemical nature of the polymer [2], however, the retention of the formulation at the site of drug application can be increased not only by an increase in viscosity, but also by a specific interaction between the polymer and the mucin layer of the precorneal tear film [3].

Mucoadhesive drug delivery systems utilize the property of bioadhesion of certain water-soluble polymers that become adhesive to mucous membranes on hydration [4] and hence can be used for targeting a drug to a particular mucus tissue (e.g. gastrointestinal. buccal, nasal, etc.) extended period of time [5].

The factors influencing mucoadhesion at these sites have already been investigated as well [6]. When adding mucoadhesive polymers to the formulation to prolong specifically the precorneal residence time of ocular dosage forms, some special features, which are characteristic for ocular drug delivery, have to be taken into consideration. When polymer dispersions are applied topically as low-viscous preparations, the polymer molecules are fully hydrated. Therefore, the hydration state of the mucoadhesive polymer has to be considered in order to evaluate the effectiveness of the mucoadhesive mechanism.

The idea of mucoadhesives is derived from the need to localize a drug at a certain site in the body. Often the extent of drug absorption is limited by the residence time of the drug at the absorption site. Particularly, in ocular drug delivery, less than 2 min are available for drug absorption after instillation of a drug solution into the eye, since it is removed rapidly by solution drainage; hence the ability to extend the contact time of a topically delivered drug would undoubtedly improve drug bioavailability.

Sodium alginate, the sodium salt of alginic acid, is a natural hydrophilic polysaccharide containing two types of monomers, beta-D-mannuronic acid (M) and alpha-L-guluronic acid (G). Alginate forms 3-dimensional ionotropic hydrogel matrices, generally by the preferential interaction of calcium ions with the G moieties resulting in the formation of an inhomogeneous gel [7]. Consequently, in situ gelling occurs because of the ionic strength of the tear fluid, also alginate has good mucoadhesive property. Sodium CMC was combined in formulation to improve viscosity and for the additive effect of mucoadhesive property. The amount of polymers included in the formulation should be low to avoid irritation and ocular disturbance. So 0.1 to 0.5% w/v sodium CMC was used in this formulation. In this concentration range sodium CMC formed a clear and stable formulation.

Sodium CMC was selected as a polymer instead of other polymers due to its better mucoadhesive capacity in comparison to that of other mucoadhesive polymers like poly (acrylic acid) (PAA), polycarbophils. PAA adhesion is very sensitive to the presence of ions, the shielding of the carboxyl group by cations present in the tear fluid diminish the interaction of PAA with the functional group on mucin [8]. Polycarbophil shows the maximum adhesion strength at pH-3, decreasing gradually with increasing in pH upto 5, above which it does not show any mucoadhesivity.

Therefore, a combination of sodium alginate with sodium CMC would be very promising for ocular administration as the mucoadhesive system. The result could be a prolonging of the contact time. In fact, combination of alginate and HPMC for the ocular delivery of gatifloxacin was already studied [9]. In that study HPMC was used to decrease the amount of alginate in the formulation. In this study HPMC was not used as it has very low mucoadhesive property.

The objective of the present work was to develop a mucoadhesive system of Gatifloxacin (GTN), a fluroquinolone derivative used in external infections of the eye using sodium alginate alone and in combination with sodium carboxymethyl cellulose, which would undergo gelation when instilled into the cul-de-sac of the eye and provide prolonged retention on the external ocular surface by a combination of gelation and mucoadhesivity of the formulation.

## Result and discussion

### Preparation of Formulations

The components of the various batches of the gatifloxacin mucoadhesive system are shown in Table 1. Initial experiments showed that increasing the concentration of sodium alginate, in preparations containing only sodium alginate, beyond 2 % w/v caused gelation upon cooling to 40 °C (during stirring). Sodium carboxymethyl cellulose combined with sodium alginate in the concentration of 0.1, 0.25, and 0.5 % w/v provided the defined fluidity of the liquid formulation.

### Evaluation of formulations

The physico-chemical properties of the prepared gatifloxacin formulations are shown in Table 2. The drug content, clarity and pH of the formulations were found to be satisfactory and the formulations were liquid at both room temperature (25–28°C) and when refrigerated (4–8°C). The two main pre-requisites of mucoadhesive systems are viscosity and gelling capacity (speed and extent of gelation). The formulation should have an optimum viscosity that will allow easy instillation into the eye as a liquid (drops). Moreover, the mucoadhesive gel should preserve its integrity without dissolving or eroding for a prolonged period of time to facilitate sustained release of the drug to the ocular tissues. All the formulations showed instantaneous gelation when contacted with the gelation fluids (STF). However, the nature of the gel formed depended upon the polymer concentration. In case of gatifloxacin formulation batche GS_1_ showed the weakest gelation, due to the presence of minimal amount of sodium alginate (0.4%).

### Rheological studies

**Fig. 1.**
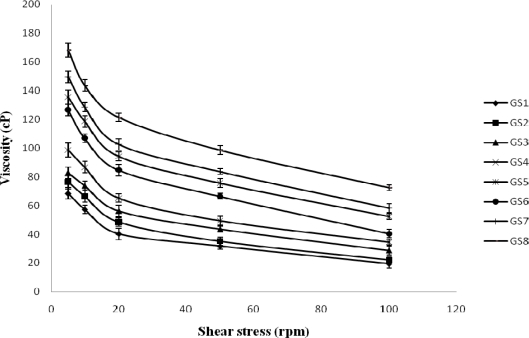
Rheological behavior of mucoadhesive system of gatifloxacin in non-physiological condition (pH – 5.0) [Values reported as mean ± SD (n = 3)]

**Fig. 2.**
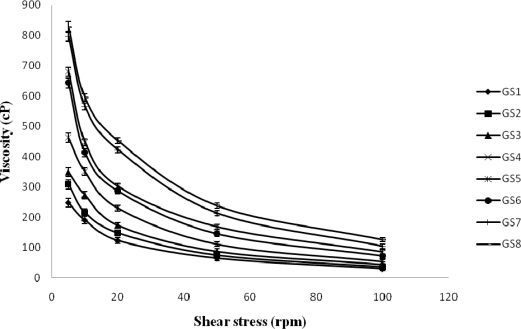
Rheological behavior of mucoadhesive system of gatifloxacin in physiological condition (pH – 7.2) [Values reported as mean ± SD (n = 3)]

The formulations exhibited pseudoplastic behaviour as evidenced by shear thinning and an increase in the shear stress with increase in the angular velocity. The administration of an ophthalmic formulation should not influence the pseudoplastic nature of the precorneal film, if they do so it should be negligible [10]. Figure 1 shows that the viscosity of the developed formulation in non-physiological condition (pH – 5.0). Figure 2 shows that the viscosity of the formulation in physiological condition (pH – 7.2). All formulations either in non-phsiological and physiological condition showed pseudo plastic behavior (viscosity that is high under the low shear rate and low under the high shear rate conditions), Which is fruitful for ophthalmic use due to the fact that the ocular shear rate is very high particularly during the blinking period [11]. The viscosity of the formulation increased with increasing concentrations of polymers.

### Evaluation of mucoadhesivity of the formulations

The viscosity of mucin colloidal dispersion is the net result of the resistance to flow exerted by individual chain segment, physical chain entanglement and the non covalent intermolecular interaction such as electrostatic, hydrogen and hydrophobic bonding [12, 13]. These interactions are the identical forces involed in the process of mucin-polymer adhesion [14]. Thus, force in a mucin bioadhesive system can be monitored by measurement of viscosity. In fact, both physical and chemical bond energies in mucin-polymer interactions can be transformed into mechanical energy or work. This work causes changes in the shape or arrangement of macromolecules and is the basis for the viscosity changes [13].

The mucoadhesive forces of different formulations of gatifloxaicn mucoadhesive systems are shown in Table 3. The mucoadhesive force was significantly (*p<*0.05) increased as the concentration of sodium alginate and sodium CMC increased over the range of 0.4 to 2% and 0.1 to 0.5 %, respectively. Formulation GS_8_ (Containing sodium alginate concentration 2%) exhibited maximum mucoadhesive strength. The result also showed that both sodium alginate and sodium CMC significantly increased the viscosity as well as the mucoadhesive property.

Sodium CMC has an abundance of hydroxyl and ether groups along their length, which are responsible for the mucoadhesive properties. Increasing the concentration of the sodium CMC in the formulation increased the bonds forming groups, thus increase the mucoadhesicve force of the formulations [15]. Mucoadhesion behavior of alginate was due to the low surface tension (31.5 mN/m) of the alginate, which is lower than the critical surface tension of the mucin coated cornea (38 mN/m), resulting in good spreading and adhesion [16].

### In vitro release

The results clearly showed that the mucoadhesive system has the ability to retain gatifloxacin in its matrix network and that premature drug release will not occur.

### Effect of sodium alginate on drug release

**Fig. 3.**
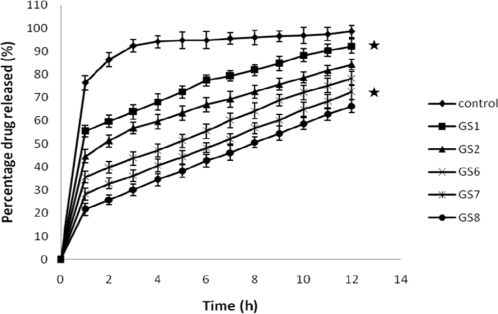
Effect of sodium alginate concentration on drug release [Values reported as mean ± SD (n = 3); * Significantly different (p < 0.001)]

Gatifloxacin release from the control system was fast and the system were completely depleted of drug within 2 hr. Sodium alginate, when incorporated as a part (0.4 % to 2 % w/v), modulated drug release significantly (*p*<0.001) (Figure 3). Alginate with a high guluronic acid content will improve the gelling properties and reduce the total polymer to be introduced into the eye. Figure 3 shows that even at lower concentrations of sodium alginate; the drug release was sustained for an extended period. Drug release seemed to slow down with an increase in sodium alginate concentration. The release was significantly (*p*<0.001) slowed when the polymer concentration increased from 0.4% to 1 and 2%, while there was not much difference between the release patterns.

### Effect of sodium carboxy methylcellulose on drug release

**Fig. 4.**
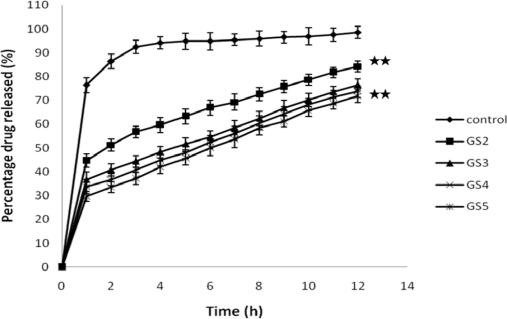
**Effect of sodium CMC concentration on drug release** [Values reported as mean ± SD (n = 3); ** Significantly different (p < 0.001)]

Figure 4 shows the effect of sodium carboxymethyl cellulose on GTN release. Sodium alginate at a concentration of 0.6% w/v was present in the formulation containing varying proportions of sodium carboxymethyl cellulose (GS_3_–GS_5_). The release of drug depends not only on the nature of the matrix, but also upon the polymer concentration. This may be due to structural reorganization of the hydrophilic polymer, sodium CMC. Increase in concentration of sodium CMC may result in increase in the gel strength of the polymer. When sodium CMC is exposed to an aqueous medium, it undergoes rapid hydration and chain relaxation to form a viscous gelatinous layer (gel layer). Comparison of the release profile of GS_2_ (containing only sodium alginate) with those of GS_3_–GS_5_ indicate that burst effect was considerably reduced. Sodium carboxymethyl cellulose, (incorporated at 0.1 to 0.5% w/v) affected the drug release significantly (*p* < 0.001).

The initial fast release of gatifloxacin from the prepared systems could be explained by the fact that these systems were formulated in an aqueous vehicle. The matrix formed on gelation was already hydrated and hence hydration and water permeation could no longer limit the drug release. A similar release pattern was reported for ciprofloxacin, wherein the initial fast release (burst effect) decreased with an increase in polymer concentration from gellan and alginate systems [17].

By reviewing the kinetic data, it could be deduced that all the prepared formulations exhibited *n* values greater than 0.5 and less than 1 indicating an anomalous or non-Fickian release suggesting a coupled erosion–diffusion mechanism for the tested gatifloxacin mucoadhesive system.

### Antimicrobial efficacy studies

#### Determination of minimum inhibitory concentration (MIC)

MIC determination was carried out using the serial dilution method. In GS_5_ formulation and pure drug sample the MIC concentration was found to be 0.15 and 0.075 μg/ml against *S.aureus* and *E.coli*, respectively. It showed that gatifloxacin retains its antimicrobial efficacy when formulated as mucoadhesive system.

#### In vitro antibacterial activity

**Fig. 5.**
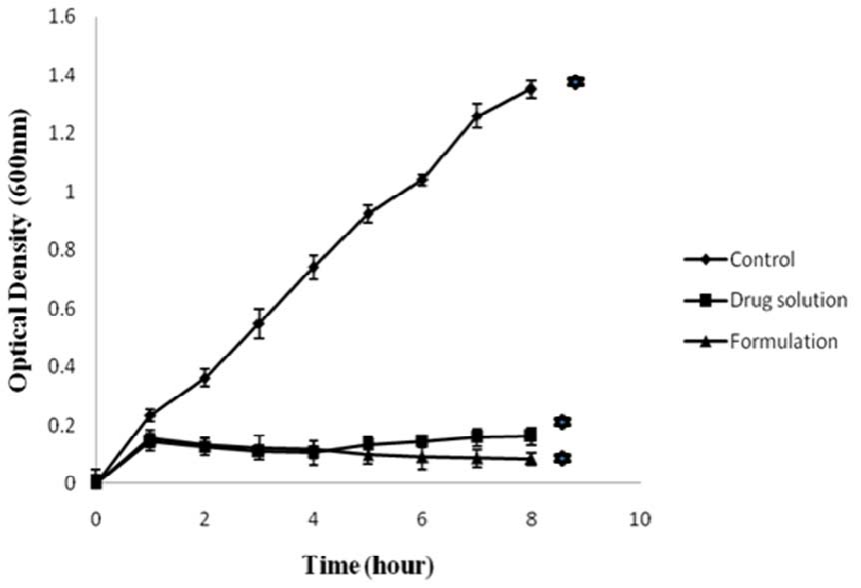
*In vitro* antibacterial activity of drug solution and formulation (GS_5_) against *E. coli*. [Values reported as mean ± SD (n = 3); * Significantly different (p < 0.001)]

**Fig. 6.**
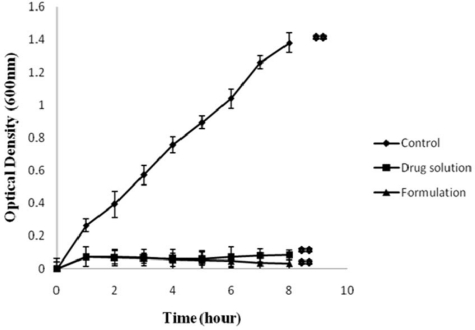
*In vitro* antibacterial activity of drug solution and formulation (GS_5_) against *S. aureus*. [Values reported as mean ± SD (n = 3); ** Significantly different (p < 0.001)]

Figure. 5 and 6 show *in vitro* antibacterial activity of free GTN and GTN mucoadhesive system against *E. coli* and *S.aureus*, respectively. A significant decrease in OD600 value was observed in comparison to control (*p*<0.001). In control set the OD600 value of *E.coli* and *S.aureus* increased with time.

In control set the number of viable organisms increased with time. Control group did not receive any antimicrobial agent. Thus under favourable condition (temperature and nutrient) the test organisms exhibited a significant growth. The growth of organisms were reduced or inhibited in presence of either in solution form or formulation which is evident from the growth curve. Although no significant difference was observed between the antimicrobial activity of the drug and formulation (*p*>0.001), a marginal increase of viable count was observed in sample tubes containing drug solution after ∼4 hrs. No increase in the viable count was seen during the 8-h incubation with formulation. These results indicate the sustained release characteristics of gatifloxacin mucoadhesive system, that released GTN inhibits bacterial growth for a longer period.

### Interaction studies

#### IR Study

**Fig. 7.**
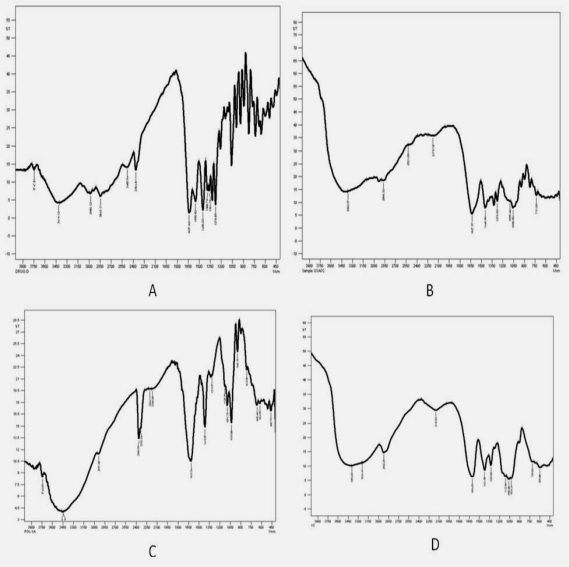
IR Spectrum solid state interaction of drug and polymers A – Gatifloxacin crystalline powder; B – powder obtained by freeze-drying the formulation (GS_5_) at 13.6 Pa and −46°C for 8 h; C – Sodium CMC; D – Sodium alginate

The IR spectrum of Gatifloxacin showed absorption bands for aromatic C-H (900-670 cm^−1^ several bands) alkane –CH_3_ (2960 cm−^1^), carboxylate (3414 cm^−1^), secondary amine (1550 cm^−1^), conjugated ketone (1637 cm^−1^). The IR spectrum of sodium alginate exhibited characteristic absorption bands for hydroxyl groups (3448 cm^−1^), carboxylate (1413 cm^−1^) and primary amine (3439 cm^−1^). The IR spectrum of sodium carboxymethyl cellulose exhibited characteristic absorption bands for carboxylate (1419, 1597 cm^−1^) and alkyl substituted ether (1155 cm^−1^). The IR spectrum of freeze dried formulation showed characteristic bands of drug, i.e., there was no interaction between drug and polymers (Figure 7).

#### DSC Study

**Fig. 8.**
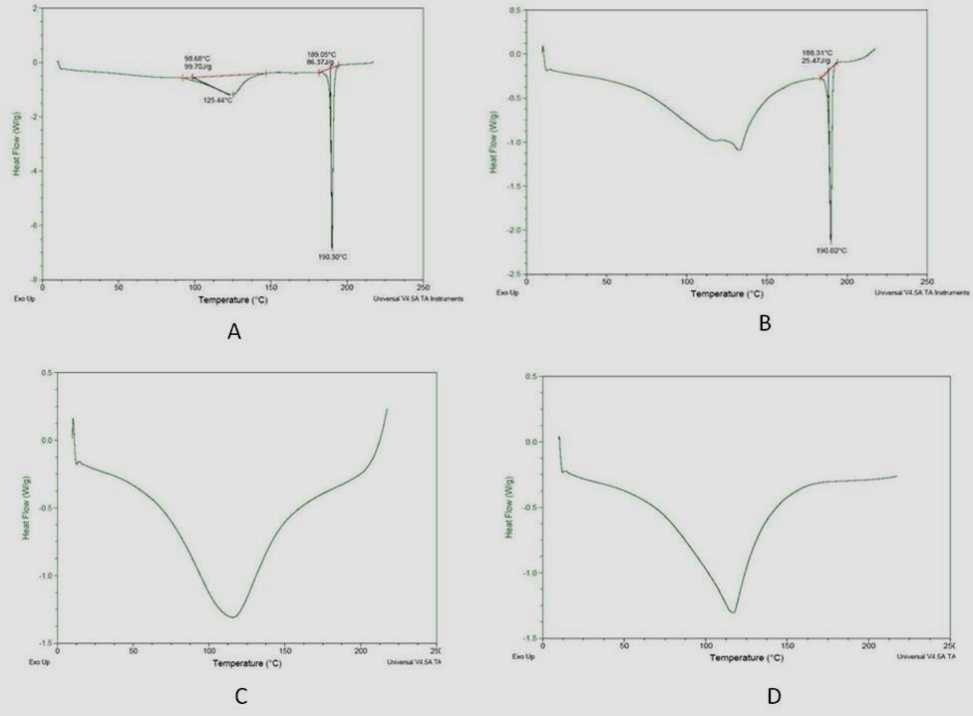
DSC endotherm of Gatifloxacin pure and freeze dried formulation (GS_5_) A – Gatifloxacin crystalline powder; B – powder obtained by freeze-drying the formulation (GS_5_) at 13.6 Pa and −46°C for 8 h; C – sodium alginate; D – Sodium CMC

DSC thermograms were recorded for pure gatifloxacin, polymers and freeze dried formulation (Figure 8). It was observed that the characteristic endotherm (corresponding to melting of the drug) did not shift appreciably, suggesting lack of any interaction between the drug and excipients.

## Experimental

### Materials

Gatifloxacin (GTN) was kindly gifted by Burgeon Pharmaceutical pvt. Ltd. (Kancheepuram, India), Sodium alginate and sodium carboxymethylcellulose were kindly gifted by Dr.Reddy’s laboratory (Hyderabad, India). Benzalkonium chloride was purchased from RFCL Ltd. (New Delhi, India). Mucin type II and Cellulose membrane were purchased from Sigma-Aldrich chemicals pvt. Ltd., New delhi, Mannitol was purchased from Sisco Research Lab. (Mumbai, India). Sodium Chloride, Sodium acetate, Calcium chloride (·2 H_2_O) and Glacial acetic acid were purchased from Merck (Mumbai, India). Sodium bicarbonate was purchased from sd fine chemical Ltd. (Mumbai, India). All other reagents were of analytical grade.

### Preparation of formulations

Sodium alginate alone and in combination with sodium carboxymethyl cellulose (Table 1) were dissolved in hot acetate buffer pH 5.0 (70°C, prepared in fresh water for injection under laminar flow) by continuous stirring at 40°C. Required quantity of gatifloxacin to give a final drug concentration of 0.3% w/v was added to the polymeric solution and stirred until dissolved. Mannitol and benzalkonium chloride were added later which as isotonicity agent and preservative, respectively. The formulations were filled in sterile 100-ml amber coloured bottles, capped with rubber closures and sealed with aluminum caps. The formulations, in their final pack were terminally sterilized by autoclaving at 121°C and 15 p.s.i. for 20 minutes. The sterilized formulations were stored in refrigerator (4–8°C) until further use.

### Evaluation of the formulations

#### Drug content uniformity

The bottle (n=3) containing the preparations were shaken for 2–3 minutes and 100 μl of the preparation was transferred aseptically to sterile 25 ml volumetric flasks with a micropipette and the final volume was made up with Simulated Tear Fluid (Sodium chloride 0.67 g, Sodium bicarbonate 0.20g, Calcium chloride (2 H_2_O) 0.008 g, Distilled water q.s. 100 ml). The concentration of GTN was determined at 287 nm (Shimadzu, UV-1601, Japan).

#### Gelation studies

The gelation studies were carried out in gelation cells, fabricated locally using Teflon^®^. The cells were cylindrical reservoirs capable of holding 3 ml of the gelation solution (STF). Within the cells located at the bottom is a 250-μl transparent plastic cup to hold the gel sample in place after its formation. 100 μl of the preparation was carefully placed into the cavity of the cup using a micropipette and 2 ml of the gelation solution was added slowly. Gelation was deducted by visual examination.

#### Rheological studies

Viscosity determinations of the prepared GTN formulations were carried out using Brookfiled DV-111+ Rheometer with spindle LV-3. The viscosity of the formulations, both in non-physiological condition (pH – 5.0) and physiological condition (pH – 7.2) were determined using a 50ml aliquot of the sample. Viscosity of the samples was measured at different angular velocities from 10 to 100 rpm with equal wait for 3 min in each rpm. The angular velocity was reversed (100 to 10 rpm). The average of two readings was used to calculate the viscosity.

#### Mucoadhesive evaluation

A simple viscometric method was used to quantify mucin-polymer bioadhesive strength [18]. Viscosities of 15% (w/v) porcine gastric mucin dispersions in STF were measured with a Brookfield viscometer in the absence (η_m_) or presence (η_t_) of different formulations at 37°C and a shear rate of 100 rpm. Viscometric measurements were performed after exactly 3 min of applying the shear force to be homogeneously distributed throughout the sample. Viscosity components of bioadhesion (ηb) was calculated from the equation, η_t_ = η_m_ + η_p_ + η_b_, where η_p_ is the viscosity of corresponding pure polymer solution. The force of bioadhesion (F) was calculated from the equation, F = η_b_.σ, where σ is the rate of shear/sec.

#### In vitro release studies

The in-vitro release of GTN from the formulation was studied through cellulose membrane using a modified apparatus [19]. The freshly prepared Simulated Tear Fluid (STF) was used as release medium. Cellulose membrane (molecular weight cut off 12,000 D, Sigma-Aldrich Chemicals, India), previously soaked overnight in the dissolution medium was tied to one end of a specifically designed glass cylinder (open at both ends). One ml of formulation (equivalent to 3mg of GTN) was mixed with 3ml of STF and placed into this assembly. The cylinder was attached to a stand and suspended in 50ml of dissolution medium maintained at 37±1°C and the membrane just touching the receptor medium surface. The dissolution medium (STF) was stirred with a star headed magnetic bead at 50 rpm. Aliquots of 5 ml volume were withdrawn at regular intervals and replaced with an equal volume of pre-warmed medium. The samples were analyzed for GTN content at 287 nm using a UV spectrophotometer (Shimadzu-1601, Japan). The in vitro release was studied for the control (without polymer) in order to compare the release profile with the mucoadhesive system of gatifloxacin.

The release mechanism of gatifloxacin from the mucoadhesive system was investigated using the following equation [20]

$$\text{Qt}/\text{Q}\alpha \;=\;{\text{Kt}}^{\text{n}}$$

where Q*t*/Q∞ is the fraction of the drug released at time *t*, *K* is a constant incorporating structural and geometric characteristics of the drug/polymer system, and *n* is the release exponent, which is indicative of the drug-release mechanism. When *n* is equal to 0.5, the drug is released from the polymer with Fickian diffusion mechanism. If 0.5 < *n* < 1, it indicates anomalous or non-Fickian release, whereas if *n* = 1, it indicates zero-order release.

### Antimicrobial efficacy studies

#### Determination of minimum inhibitory concentration (MIC)

To carry out MIC test the serial dilution method was employed [21]. *Staphylococcus aureus* (ATCC 25923) and *Escherichia coli* (ATCC 25922) were used as gram-positive and gram-negative organisms, respectively.

The stock solution of gatifloxacin in both standard and test were prepared in the concentration of 3 mg/ml in acetate buffer (pH 5.0). 15 sterile test tubes were arranged in a rack and numbered from 1 to 15. From the stock solution serial dilutions were prepared using Lubria broth (LB) to give concentrations of 76.5μg/ml to 0.02μg/ml placed in 1^st^ tube to 13^th^ tube. One tube was considered as positive control and another tube as negative control. Following subcultures from frozen stock, 10μl broth of the standard *S. aureus* and *E.coli* containing 1×10^6^ CFU/ml strains were inoculated in all the test tubes except in negative control and incubated at 37°C for 24h to observe the growth. The tubes were observed for inhibition of growth and MIC was determined.

#### In vitro antibacterial activity

The antimicrobial sensitivity of the formulation was examined *in vitro* by standard procedure [21]. 1ml of the sample (GS_5_) was mixed with 100ml of STF solution for gelation. 1ml of the above solution was added to a set of test tubes containing cultures of standard *E. coli* and *S. aureus* (1×10^6^ CFU/ml) in LB broth. In another set of experiment drug solution equivalent to 30μg was used. The test tubes were incubated at 37±0.2°C, with continuous shaking. At 1, 2, 3, 4, 5, 6, 7 and 8h time intervals, bacterial growth was determined by reading optical density at 600 nm (OD 600). Positive and negative controls were maintained throughout the study for comparison. The entire test was performed in triplicate and the values correspond to average ± S.D.

### Interaction studies

Interaction studies were conducted by FT-IR and DSC to investigate any interaction between drug and excipients.

#### IR Study

The drug or polymer or powder obtained by freeze-drying the formulation (GS_5_) at 13.6 Pa and −46°C for 8 h mixed with solid potassium bromide (KBr). The mixture was then pressed into a very thin pellet. The pellets were placed in the holder directly in the IR laser beam. Spectra were recorded using Shimadzu FTIR-8400s loaded with IR solution version 1.2 software. IR spectra of drug and polymer physical mixture were compared with IR spectra of pure drug for any major interaction.

#### DSC Study

DSC studies of pure drug, polymer and powder obtained by freeze-drying the formulation (GS_5_) at 13.6 Pa and −46°C for 8 h were done. The samples (2.5mg) were taken in solid state in the pan and were compressed with a high-pressure press. DSC of the samples was performed using Modulated DSC systems (Q 1000 TA equipped with software Pyris 6.0). Thermal scanning was carried out at a rate of 10°C min^−1^ (from 0–300°C) and nitrogen purge 35 mL/min.

## Conclusion

Gatifloxacin was successfully formulated as a mucoadhesive system using a combination of sodium alginate with sodium carboxymethyl cellulose. The formulated systems provided sustained release of the drug over a 12-hr period in vitro. Significant reduction in total bacterial count was observed between control and treatment groups with the two organisms, viz. *S. aureus* and *E.coli*. The developed formulation is a viable alternative to conventional eye drop due to its ability to enhance bioavailability through its longer precorneal residence time and ability to sustain release of the drug. Also important is the ease of administration afforded and decreased frequency of administration resulting in better patient compliance.

## Figures and Tables

**Tab. 1.** Components of mucoadhesive system of gatifloxacin

**S.No**	**Batch code**	**Gatifloxacin (% w/v)**	**Sodium alginate (% w/v)**	**NaCMC (% w/v)**
1	Control	0.3	–	–
2	GS_1_	0.3	0.4	–
3	GS_2_	0.3	0.6	–
4	GS_3_	0.3	0.6	0.1
5	GS_4_	0.3	0.6	0.25
6	GS_5_	0.3	0.6	0.5
7	GS_6_	0.3	0.8	–
8	GS_7_	0.3	1	–
9	GS_8_	0.3	2	–

Mannitol 5% was used as isotonic agent and benzalkonium chloride 0.02% was used as a preservative.

**Tab. 2.** Physico-chemical properties of the prepared Gatifloxacin mucoadhesive systems

**Batch Code**	**Drug content uniformity (% ± S. D.)**	**Gelling capacity in STF**	**pH**
Control	99.58 ± 1.05	−	5.1
GS_1_	99.58 ± 0.25	+	5.3
GS_2_	98.91 ± 0.71	++	5.2
GS_3_	99.32 ± 0.95	+++	5.3
GS_4_	98.73 ± 1.03	+++	5.3
GS_5_	99.51 ± 0.67	+++	5.2
GS_6_	99.45 ± 0.54	+++	5.3
GS_7_	98.64 ± 0.62	+++	5.4
GS_8_	100.20 ± 0.55	+++	5.3

+…Gels after few minutes; ++…Gels immediately but remains for a few hours (less stiffer); +++…Gelation immediate and remains for extended periods and formed gels are stiffer.

**Tab. 3.** Viscosity of formulation (η_p_), component of mucoadhesion (η_b_) and the force of mucoadesion (F) in stimulated tear fluid (STF)

**Formulation**	**Polymer concentration (W/V, %)**		**η_p_ (cPs)^a^**	**η_b_ (cPs)^a^**	**F (dyne/cm^2^)^a^**
	**Sodium alginate**	**Sodium CMC**			
GS1	0.4	–	28.7 ± 2.61	10.2 ± 2.10	17.03 ± 2.40*
GS2	0.6	–	35.4 ± 3.20	16.3 ± 2.65	27.22 ± 2.60
GS3	0.6	0.1	43.8 ± 2.54	28.2 ± 3.12	47.10 ± 3.20
GS4	0.6	0.25	55.6 ± 3.41	37.5 ± 3.51	62.63 ± 3.45*
GS5	0.6	0.5	84.3 ± 2.61	52.6 ± 4.21	87.84 ± 4.25*
GS6	0.8	–	73.5 ± 4.21	43.3 ± 2.50	72.31 ± 4.61
GS7	1	–	106.8 ± 5.61	57.7 ± 3.60	96.36 ± 3.54
GS8	2	–	125.7 ± 4.36	63.6 ± 4.20	106.2 ± 5.70

a Values reported as mean ± SD (n = 3);

* Significantly different (p < 0.05)
